# Complete Genome Sequence of *Lactobacillus plantarum* 10CH, a Potential Probiotic Lactic Acid Bacterium with Potent Antimicrobial Activity

**DOI:** 10.1128/genomeA.01398-17

**Published:** 2017-11-30

**Authors:** Nancy M. El Halfawy, Moustafa Y. El-Naggar, Simon C. Andrews

**Affiliations:** a Department of Botany and Microbiology, Faculty of Science, University of Alexandria, Alexandria, Egypt; b School of Biological Sciences, University of Reading, Reading, United Kingdom

## Abstract

*Lactobacillus plantarum* 10CH is a bacteriocin-producing potential probiotic lactic acid bacterium (LAB) strain isolated from cheese. Its complete nucleotide sequence shows a single circular chromosome of 3.3 Mb, with a G+C content of 44.51%, a 25-gene plantaricin bacteriocin gene cluster, and the absence of recognized virulence factors.

## GENOME ANNOUNCEMENT

*Lactobacillus plantarum* is an inhabitant of the human gastrointestinal tract (1) and is used as a probiotic, since it is generally regarded as safe, confers beneficial health effects in humans, and exhibits antimicrobial activity against microbial pathogens (2, 3). From 50 novel lactic acid bacterium (LAB) isolates, the extracellular products of *L. plantarum* 10CH (isolated from cheese) gave the highest antimicrobial activities against a panel of 13 indicator strains, including *Listeria monocytogenes*, *Staphylococcus aureus*, *Enterococcus faecium*, *Enterococcus faecalis*, and *Salmonella enterica*, characteristic of bacteriocin production.

Genomic DNA of *L. plantarum* 10CH was isolated using a GeneJET purification kit and assessed using a NanoDrop ND-1000 spectrophotometer and by electrophoresis. Genome sequencing was performed by Microbes NG (University of Birmingham, UK) using MiSeq and HiSeq 2500 platforms (Illumina, UK). The genome sequencing yielded 968,127 reads, with a median insert size of 489 bases and 123-fold coverage of the genome. The reads were trimmed using Trimmomatic (4) by identification of adapter sequences, and the quality of the trimmed reads was assessed using in-house scripts combined with the bwa-mem software (5). These reads were *de novo* assembled with SPAdes software version 3.7.0 (6), yielding 48 large contigs (>1,000 bp). The quality of the genome assemblies was assessed using the Quality Assessment Tool for Genome Assemblies (QUAST) (7). The draft genome was mapped against the published reference genome of *L. plantarum* WCSF1 (GenBank accession number AL935263), which was found to be the closest neighbor by the NCBI server using the CONTIGuator mapping tool (8). Based on the Artemis Comparison Tool view, contigs were arranged, and the intrascaffold gaps were then determined. Thirty-two pairs of PCR primers were designed to fill the putative gaps between the contigs, and the PCRs were carried out using high-fidelity CloneAmp HiFi PCR premix (TaKaRa, Japan). The resulting amplicons were analyzed by gel electrophoresis and sequenced by Eurofins Genomics. The sizes of the amplified gaps ranged from 900 bp to ~5.5 kb. SeqBuilder (Lasergene) was used to fill the gaps using the resulting sequences of the amplified PCR products.

The sequencing results were consistent with the presence of a single circular replicon of 3,311,056 bp (no plasmids were found). The complete genome was annotated using the NCBI Prokaryotic Genome Annotation Pipeline (PGAP) and the Pathosystems Resources Integration Center (PATRIC) Web server (9, 10). This revealed a total of 3,192 protein-coding genes, along with 6, 5, and 5 5S, 16S, and 23S rRNA genes, respectively, 67 tRNA genes, and 100 pseudogenes. Four prophage loci ranging in size from 26 to 51 kb were also identified in the chromosome using the PHAST Web server (11). Five adjacent plantaricin gene clusters were predicted (*plnRLKJ*, *plnMNOP*, *plnABCD*, *plnIFE*, and *plnGHTUVW*) using Vector NTI *Express* (Invitrogen) and are responsible for the production of plantaricins A, EF, and JK. Similar bacteriocin clusters are found in *L. plantarum* ST-III (GenBank accession number CP002222), C11 (X94434), and V90 (FJ809773). No classical virulence genes were identified in the *L. plantarum* 10CH genome, which, together with its strong and broad-spectrum antimicrobial activity, indicates its potential suitability as a probiotic strain.

### Accession number(s).

The complete genome sequence of *L. plantarum* 10CH has been deposited at GenBank with the accession number CP023728.
